# Identifying Crystal Structure of Halides of Strontium and Barium Perovskite Compounds with EXPO2014 Software

**DOI:** 10.3390/ma18010058

**Published:** 2024-12-26

**Authors:** Jorge A. Perez Franco, Antonieta García Murillo, Felipe de J. Carrillo Romo, Issis C. Romero Ibarra, Arturo Cervantes Tobón

**Affiliations:** 1 Instituto Politécnico Nacional CIITEC, Azcapotzalco, Mexico City 02250, Mexicofcarrillo@ipn.mx (F.d.J.C.R.); 2 Instituto Politécnico Nacional-UPIITA, Mexico City 02580, Mexico; 3 Instituto Politécnico Nacional ESIQIE, Mexico City 07700, Mexico

**Keywords:** EXPO2014, band gap, lead-free perovskite, strontium perovskite, barium perovskite

## Abstract

The synthesis of ethylamine-based perovskites has emerged to attempt to replace the lead in lead-based perovskites for the alkaline earth elements barium and strontium, introducing chloride halide to prepare the perovskites in solar cell technology. X-ray diffraction studies were conducted, and EXPO2014 software was utilized to resolve the structure. Chemical characterization was performed using Fourier transform infrared spectroscopy, photophysical properties were analyzed through ultraviolet–visible spectroscopy, and photoluminescence properties were determined to confirm the perovskite characteristics. The software employed can determine new crystal structures, as follows: orthorhombic for barium perovskite CH_3_CH_2_NH_3_BaCl_3_ and tetragonal for strontium perovskite CH_3_CH_2_NH_3_SrCl_3_. The ultraviolet–visible spectroscopy data demonstrated that a temperature increase (90–110 °C) contributed to reducing the band gap from 3.93 eV to 3.67 eV for barium perovskite and from 4.05 eV to 3.84 eV for strontium perovskite. The results exhibited that new materials can be obtained through gentle chemistry and specialized software like EXPO2014, both of which are capable of conducting reciprocal and direct space analyses for identifying crystal structures using powder X-ray diffraction.

## 1. Introduction

Perovskite solar cells have become one of the most promising technologies due to their high energy conversion efficiency, low production cost, and easy manufacturing process. Perovskite materials are considered as one of the eminent materials for new-generation PV technology because of their unique properties, such as a high electron mobility, high carrier diffusion length, ambipolar charge transport behavior, high absorption coefficient due to s-p antibonding coupling, low exciton binding energy, high photoluminescence quantum efficiency, high carrier lifetime, tunable bandgap, great structural defect tolerance, and amiable grain boundary effect [1,2].

The use of perovskite light collectors in solar cells represents a great advance. They have optimal properties that enable them to be used as light absorbers, such as their band gap (ranging from 1.2–1.8 eV); their large absorption coefficients in the visible, ultraviolet, and infrared spectrums; and their high carrier mobility. The band gap expands their optoelectronic applications [3,4]. However, it brings many obstacles towards commercialization in the meanwhile. Firstly, the chemical bonding between ions results in a limited stability. Perovskite materials, even those with a high purity, are inclined to degrade under ambient conditions. Even at a very low concentration level, the presence of moisture and oxygen has a tremendous effect on perovskite stability [5,6].

The high performance of lead-based perovskite solar cells arises from their ideal band gap, which is the amount of energy required to generate an electron–hole pair. If the striking photon has more energy than this, a hole pair is generated, and the extra energy is converted to heat in the solar cell. The band gap determines which energy particles (photons) the solar cell can absorb in sunlight. If the band gap is too large, many photons do not have enough energy to make the electrons jump. If the band gap is too small, excess energy will be wasted. Perovskite solar cells account for losses induced by the thermal irradiation of a cell, the recombination of electron and hole carriers, and sunlight spectrum losses due to the Shockley–Queisser efficiency limit, also affecting the nonradiative recombination of excitons, including mediation by phonons, admixtures, defects, or other carries along the Auger recombination scheme [7,8,9].

The crystal structures of various types of perovskite halide compounds possess an ideal cubic or tetragonal shape with an ABX_3_ stoichiometry, where A is an organic cation, B is a metal cation, and X is a halide anion. The B cation is octahedrally coordinated with the X anions, with the octahedra sharing corners to form the basic building block of the perovskite structure. Their optical properties can be adjusted (i.e., E_g_) to improve their performance through the exchange of cations (at sites A and B) or by manipulating the type of halide at site X [10].

The BX_6_ octahedra are connected in a three-dimensional, shared-corner configuration, with the A cation embedded in the space between the adjacent BX6 octahedra, which neutralizes the structural charge. Cation A fills 12 coordinated cavities formed by the BX_3_ network and is surrounded by 12 equidistant anions found in the center of the network [11,12,13,14].

However, in many perovskites, distortions are observed because there are differences between the ionic radii of the A and B cations, which causes the X anions and the B cations to move, giving rise to tilts and rotations in the octahedral coordination. The unit cell can be modified by partial substitutions of the B cations to produce double perovskites of the A_2_BB’X_6_ type [15].

The most-studied perovskite absorbent materials are based on lead halide (hybrid) perovskites that possess an ABX_3_ structure, where A is a monovalent organic cation (e.g., methylamine (CH_3_NH_3_^+^, MA^+^) and formamidinium (CH(NH_2_)^2+^, FA+)) or an inorganic cation (e.g., K^+^, Rb^+^, and Cs^+^) and B is a divalent Pb^2+^ metal cation. The X site of the perovskite structure is occupied by halide anions (X = Cl^−^, Br^−^, and I^−^), with the CH_3_NH_3_PbX_3_ perovskite being the most used (A = MA, B = Pb, X = Br^−^, I^−^, Cl^−^) [16,17].

Their potential for use in solar cells corresponds to the organic anion (at the A site) of methylammonium (MA), formamidinium (FA), or another precursor such as dimethyl ammonium (DA), azetidine (AZ), or ethylammonium (EA), with lead as the metal cation at the B site and a halide—either iodide, chloride, bromide, or fluoride—at the X site [18,19]. Cation B, therefore, can be an element with an ionic radius similar to that of Pb^2+^, such as Sn^2+^, Bi^2+^, Ge^2+^, Sb^3+^, or Mn^2+^ or an alkaline earth element such as Ca^2+^, Sr^2+^, or Ba^2+^. Exchanging the lead ion for one of these results in a less toxic, lead-free perovskite for converting visible light into electricity. Some lead-free perovskite compositions often significantly reduce solar cell efficiency, e.g., a wide band gap [8,20,21,22].

Among the elements mentioned above for replacing the lead metal cation at site B, strontium and barium are preferred due to the sizes of the Sr^2+^ (132 pm) and Ba^2+^ (149 pm), which are very similar to that of the Pb^2+^ ion (133 pm). Alkaline earth elements are also considered to be interesting candidates for replacing the lead ions without altering the crystal structure at room temperature. Methylammonium (a substance added by DOF Agreement 11-23-2009) (217 pm), the ion commonly used as an organic cation at site A, can be replaced by another precursor, since it is regulated in many countries, so exchanging it for ethylammonium (274 pm) may hold promise [23,24].

Determining the crystal structure is crucial to obtaining new materials; it defines the properties of the materials, such as their absorption, conductivity, and magnetism [25].

Establishing the structure using X-ray diffraction normally consists of the following four steps:

(a) Indexing the Bragg reflections.
(b) Determining the space group.
(c) Resolving the crystal structure.
(d) Refining the structural model.

In recent work, machine learning models have proved capable of rapidly identifying known compounds (or their symmetries) from their X-ray diffraction patterns. The data from experimental XRD patterns are analyzed in terms of qualities such as shape, peak height, and position. Using these descriptors, matching the XRD test patterns to known patterns in XRD databases makes it possible to identify a compound of interest. Direct space and reciprocal space methods, statistical methods, and single-crystal growth have been used to obtain data on the crystal symmetry of new materials [26,27].

Establishing the crystal structure of materials in powder form has become widespread, since current diffraction analysis is based on advanced computational tools that use methodologies and programs that can calculate and predict the structure of a material. The tolerance factor method (based on a new tolerance factor), the Vienna Ab initio Simulation Package (VASP), Material Studio 6.1 software based on density functional theory (DFT), and the density of states function (DOS) are some examples of tools and methods for calculating structures [28,29].

Advances in resolving crystal structures using X-ray diffraction are attributable to the availability of several computer programs (some free), such as the EXPO2014 program, which can facilitate the process, from determining the space group to arriving at a complete solution, including refinement of the structure [30,31]. EXPO2014 has been updated with several graphical and computer tools to analyze crystal structures of different sizes and complexity. Some diffraction patterns have peaks associated with impurities that correspond to one or more crystalline phases and that cannot be eliminated by purification techniques, and, thus, can give rise to unexpected phases. EXPO2014 can manage patterns of multiple phases, use direct space or reciprocal space methods, or both, and apply several software algorithms to define structures.

Therefore, the current study sought novel alternatives in lead-free perovskite materials that could preserve the optoelectronic properties of Pb. Replacing Pb^2+^ with simple and mixed elements was investigated. At site A, the organic methylammonium ion was replaced with ethylammonium; at site B, Pb was replaced with alkaline earth elements (Sr and Ba); and at site X, the halogen ion Cl^−^ was used. Only a few studies have reported experiments in which Pb was replaced by two non-toxic elements and methylammonium by a precursor alternative [32].

## 2. Materials and Methods

**Figure 1:**
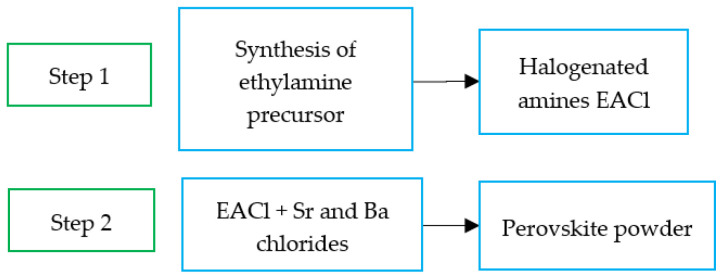
Steps for synthesizing the strontium and barium ethylamine chloride perovskites.

To synthesize strontium and barium ethylammonium chloride perovskite by the sol-gel method, a wet chemistry procedure was used, equivalent to the procedure for obtaining lead methyl ammonium chloride perovskite. The first step in the synthesis of Sr and Ba perovskites involved obtaining ethylammonium chloride (EACl). In the second stage, barium and strontium chlorides were added to the ethylamine chloride. The general process for obtaining the perovskites is schematized in Figure 1.

### 2.1. Synthesis of Ethylammonium Chloride

For the synthesis of ethylammonium chloride (CH_3_CH_2_NH_3_Cl), 10 mL of ethylamine (CH_3_CH_2_NH_2_ 66–72% Sigma-Aldrich, Burlington, MA, USA) was added to a 50 mL flask with 10 mL of hydrochloric acid (HCl at 36.7% purity) in a bath of dry ice for 60 min, with stirring at 5 rpm to avoid the volatilization of the chlorine ions. Any hydrogen chloride formed would immediately react with excess ethylamine to give ethylammonium chloride, and this induced the following chemical reaction:

(1) $$CH_{3}CH_{2}NH_{2}+HCl\to CH_{3}CH_{2}NH_{3}$$

Subsequently, the solvent was evaporated using a rotary evaporator for 120 min at 70 °C to obtain the halogenated amine, identified by its characteristic milky white appearance.

### 2.2. Strontium and Barium Perovskite Synthesis

The preparation of the perovskite precursor solution for both elements consisted of mixing 6 mmol of EACl (CH_3_CH_2_NH_3_Cl) and 6 mmol of barium chloride (BaCl_2_) or strontium chloride (SrCl_2_) in a flask at 80 °C, then adding 10 mL of deionized water under stirring for 2 h. The molar ratio used was 3:1 in 6.0 mL of dimethylformamide (HCON(CH_3_)_2_ 99.8% Karal, Burlington, MA, USA), with 1 mL of ethanol as solvent. The resulting solution was stirred for 2 h at 5 rpm. Finally, the Sr and Ba perovskites (CH_3_CH_2_NH_3_BaCl_3_ and CH_3_CH_2_NH_3_SrCl_3_, respectively) were dried at 90, 100, and 110 °C for 60 h; the samples were prepared as powders. Under UV excitation at 365 nm, the Ba and Sr perovskites presented a semi-transparent nature and blue emission.

### 2.3. Experimental Techniques

To study the characteristics of the strontium and barium perovskites for the functional groups, FT-IR was used. The equipment used for the FT-IR analysis was a Perkin-Elmer model spectrum 65 (New Haven, CT, USA) in the range from 400 to 4000 cm^−1^ whit a speed of 5 scans per minute.

The resolution of the crystal structure, the positions of the located atoms, the cell parameters, and the space group were determined using the EXPO2014 software. X-ray diffraction patterns were recorded at room temperature with a Rigaku Miniflex 600 diffractometer (Urbana, IL, USA), at an angular range of 2θ = 5–90°, a CuKα of 1.54 Å, and a step size of 0.0140.

The optical properties of the perovskites were subsequently analyzed using absorption, photoluminescence, and spectroscopy, which enabled us to obtain the spectra for the perovskites of strontium and barium on a Hitachi F7000 FL spectrometer (Chiyoda, Tokyo, Japan) with a 150 W xenon lamp and a R928F photomultiplier tube. The absorption spectra were in a range from 200 to 450 nm, with an emission wavelength $\left({ʎ}_{Em}\right)$ of 470 nm, and the emission spectra were measured in the range from 400 to 900 nm, with $\left({ʎ}_{Ex}\right)$ of 423 and 381 nm [33]. The output slit was set at 2.5, as was the input slit, and the wavelength scanning speed was 1200 nm/min.

To obtain the band gaps (E_g_) of the strontium and barium perovskites, photoluminescence absorption studies were complemented by UV–Vis spectrometric analysis in a range of 200–800 nm, using an Agilent Cary Series UV–Vis–NIR spectrophotometer (Santa Clara, CA, USA).

### 2.4. Crystal Structure Resolution

To carry out the structural characterization process with direct methods (DMs), the procedures in the EXPO2014 software were applied to the X-ray diffraction patterns of the strontium and barium ethylamine chloride perovskite powders. The software compared the observed powder diffraction patterns with the calculated patterns corresponding to the crystal structure model under study. The structure of the perovskites warranted this because they corresponded to metal–organic molecular structures, they had the advantage of not being very sensitive to the experimental resolution of the collected powder diffraction pattern, and they were able to also solve structures with quite a large number of non-hydrogen atoms in the asymmetric unit [34]. Therefore, this procedure was chosen for this comparison. It was not advisable to use reciprocal space (RS) methods because they are single-crystal-like processes and can be carried out with poor background estimates, a limited resolution, and overlapping of the peaks, leading to errors in the experimental structure modules. For this study, space groups, the contents of asymmetric units, and experimental resolutions were analyzed through DMs. Finally, a new test method was used, because these perovskites are new variants that are not reported in the Crystallography Open Database (COD) and the International Center for Diffraction Data (ICDD), since their synthesis differs from the classic synthesis of lead perovskite using methylamine.

Using the EXPO2014 software, the crystal structures of the barium and strontium perovskites in the crystalline powders were determined. To use the software, the molecular formula of the material and the powder X-ray diffraction data must be known [35]. The steps followed by the program to resolve a crystal structure were as follows: peak search, indexed, cell reduction and conventional cell calculations, unit cell parameter refinement, 2θ shift, and list of plausible cells to obtain structure model optimization.

To apply DMs using the EXPO2014 software on the diffraction data from the powders, the decomposition of the complete experimental powder pattern was required to determine the unique diffraction intensities for each Bragg reflection in the 2θ range. To calculate the integrated intensities, the program used the Le Bail algorithm [36]. Afterward, the normalization of the integrated intensities was carried out by statistical analysis to suggest the presence or absence of the inversion center, identify the possible presence and type of pseudotranslational symmetry, and detect preferred orientation effects. Also, a calculation of the invariant relationships of the structure was performed; the invariant statistical reliability of the structure was considered.

Several phases were generated from the above, approximately twenty of which were ranked and selected based on the CFOM’s available merit figure. The set of phases corresponded to (a) the largest CFOM and (b) the density of the electron map, which resulted in improving the structure model by appropriately weighing it with suitably weighted least squares (wLQs). Each structural model was refined using automatic Fourier squares (LSQ) to simplify determining the structure. The direct space method (DM) usually generates several phases that are classified according to their figure of merit, since EXPO2014 correlates with the greater merit figure [37,38].

The indexing process was performed using a graphical interface (capable of uploading a powder diffraction pattern file) to define the unit cell parameters (a, b, c, α, β, and γ) and assign the Miller indices (hkl or h) at each interplanar spacing (dh). This step enabled the reconstruction of a three-dimensional elemental cell using information about the dh values extracted from the experimental one-dimensional pattern. This indexing process was carried out with the indexing programs N-TREOR09, DICVOL06, and McMaille, giving a minimum figure of merit. The reliability of the indexing was evaluated using M20 FOMNew, M20, and McM20 as the numerical figures of merit appropriate for each process.

To identify the space group, EXPO2014 used a probabilistic procedure to recognize extinction symbols (from lists of possible space groups). Statistical information about the normalized intensities (extracted by the Le Bail method) was used to calculate the probability of each extinction symbol’s compatibility with the crystal system suggested by the indexing process of the EXPO2014 program. Thus, an appropriate weight was associated with the intensity of each reflection, reflecting uncertainty about its estimation, and by this means, errors in the experimental intensities were avoided and prevented from compromising the identification of the extinction group and, in turn, the space group [39].

To complete the model of the structures, EXPO2014 improved the quality of the Fourier map obtained at the end of Direct Methods and can use different optimization strategies, as follows:

Fourier recycling (wLSQ) is based on minimizing the sum of the weighted differences between the observed and calculated intensities. This rules out spurious atomic positions and readjusts out-of-place positions toward true positions, reducing the contribution effects of overlapping reflections and compensating for the low accuracy of the intensities. The procedure consists of eight blocks, with each block involving combined-cycle least squares weighting with $\left(2\left|F_{0}\right|-\left|F_{C}\right|\right)$ for the Fourier calculations [40].

Resolution bias minimization (RBM) reduces the scattering factor of light atoms, which can cause the experimental resolution to sometimes be far from the atomic resolution. This procedure is based on a resolution-dependent correction function, which modifies the Gaussian functions to improve the peaks in an experimental pattern. This reduces the electron density map errors caused by a limited experimental resolution [41].

Finally, the COVMAP procedure is used, which modifies the electron density based on the concept of covariance between points in the experimental pattern of an electron density map, following the procedure of the structural model provided by RBM, which uses the covariance approach. Since it modifies the electron density map of the experimental pattern, at the same time, the improved model is subjected to Fourier least squares analysis (wLSQ), resulting in new atomic localization positions based on covariance. COVMAP modifies the resulting model and processes it cyclically in the other two steps (wLQS and RMB), depending on the complexity of the structure. The number of cycles depends on the complexity of the structure, and the procedure should be performed when RBM and wLQS fail to work through DMs [42].

## 3. Results and Discussion

### 3.1. Structural Analysis

At the B site, the metal cations (Ba and Sr) are deformed due to the lengths and angles of the bonds between the B atoms at this volume, and the contraction is due to the constriction of the network in three directions.

The barium and strontium ions are larger than the halogen ions and the organic ethylamine cation, and this results in orthorhombic and tetragonal structures, respectively, suggesting that the perovskites may be arranged in layers. These structures indicate that the ethylamine cations do not have a spherical symmetry. Given that Sr^2+^, Ba^2+^, and Pb^2+^ ions have almost the same charge and radius, it is reasonable to assume the halogen structure would remain if the lead were replaced by strontium or barium.

Barium ethylamine chloride crystals and strontium have perovskite structures and undergo structural transitions upon heating. Above 150 °C [43,44] during drying, the chlorides volatilize and act to control crystallization.

For the Ba and Sr perovskite models, the ethylamine (EA) ions (CH_3_CH_2_NH_2_) have polarity and are found on the faces and in the centers of the unit cells [45].

**Figure 2:**
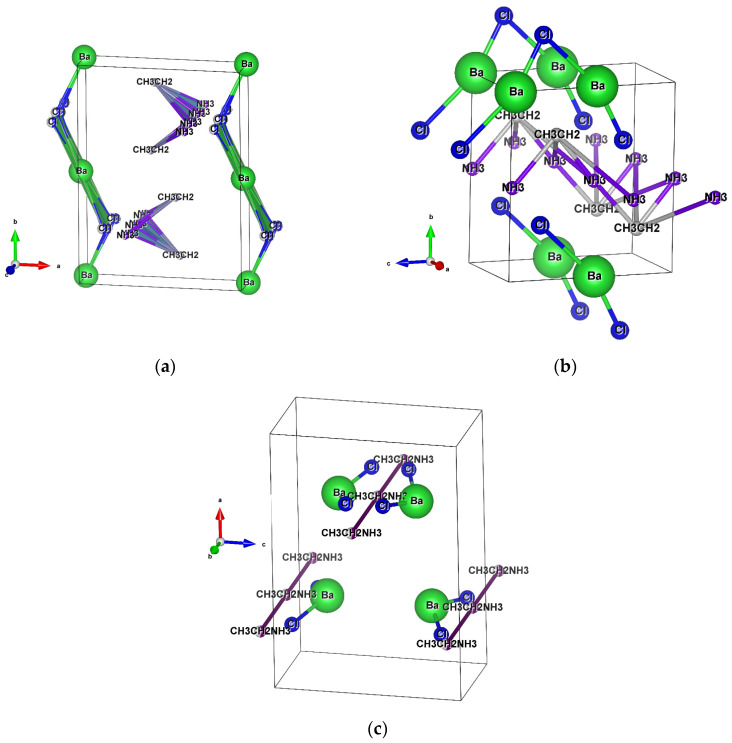
Crystal structure visualization by EXPO2014 of barium perovskite (CH_3_CH_2_NH_3_BaCl_3_) at different temperatures: (**a**) at 90 °C, (**b**) at 100 °C, and (**c**) at 110 °C, with an orthorhombic structure. The structural models shown were drawn with VESTA software (Ver. 3.5.8).

**Figure 3:**
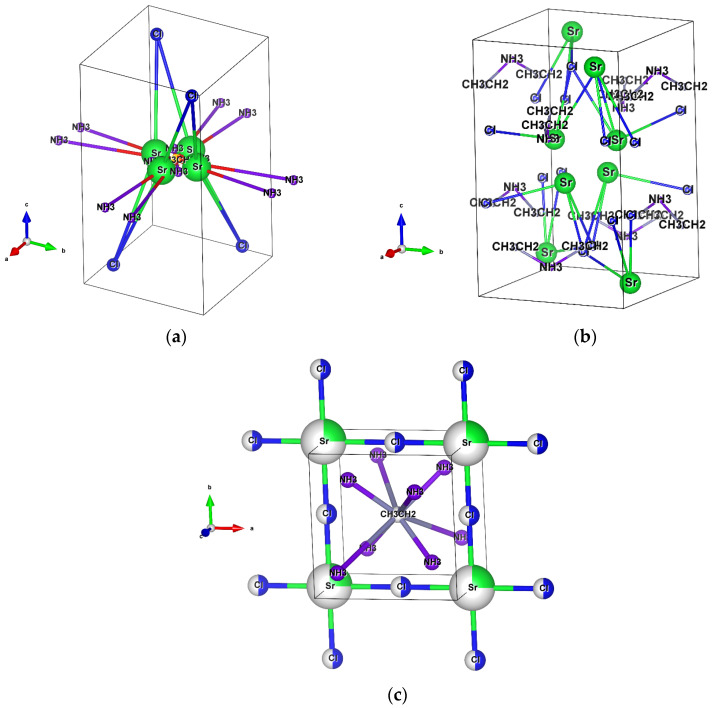
Crystal structure visualization by EXPO2014 of strontium perovskite (CH_3_CH_2_NH_3_SrCl_3_) at different temperatures: (**a**) at 90 °C, (**b**) at 100 °C, and (**c**) at 110 °C, with a tetragonal structure. The structural models shown were drawn with VESTA software.

At different temperatures (90, 100, and 110 °C), the barium perovskites form orthorhombic structures, and the strontium perovskites are tetragonal, as shown in Figure 2 and Figure 3 and Table 1 and Table 2. In the orthorhombic crystal of CH_3_CH_2_NH_3_BaCl_3_ (see Figure 2a–c), the atoms are arranged in different configurations as the temperature increases. In the barium perovskite crystal, the Cl^−^ ions are linked to the barium ions in the bases and the centers of the cells and create disorder at three different positions.

The occupancy of the CH_3_CH_2_ and NH_3_ atoms by ethylamine is detected at a position near the center of the unit cell. Because of the lattice angles, the volume of the system is affected, and the rotation and displacement of the A-cations lead to distortions and anharmonic vibrations in the whole perovskite structures [46]. This ethylamine cation is introduced into the perovskite to passivate the substitution defect by the methylamine cation. In the Sr perovskite, the CH_3_CH_2_ and NH_3_ atoms are found also in the central part of the cell (Figure 3a,c). These ions intercalate the layers in the structure of Sr^2+^ and Cl^−^, such that the chlorine is bound to the strontium ions and allows the structure to share faces in the cell. There is a weak bond between the hydrogen atoms and the anion (H^+^-Cl^−^), causing structural distortions and variations in volume [47,48].

In the strontium perovskite at 100 °C, a similar situation occurs (Figure 3b), in which the bonding of the CH_3_CH_2_, NH_3_, and Sr^2+^ with the Cl^−^ ions is not very stable, and the atoms have a structural disorder, dispersed within the unit cell. This may be due to the arrangement of atoms by the temperature transition.

The structure of CH_3_CH_2_NH_3_SrCl_3_ at 110 °C, seen in Figure 3c, can be described as pseudo-tetragonal, showing a better atomic arrangement than that of other strontium perovskites, indicating that structural transitions take place upon heating without a loss of chlorine ion properties.

### 3.2. Structural Analysis of Experimental Patterns by EXPO2014

**Figure 4:**
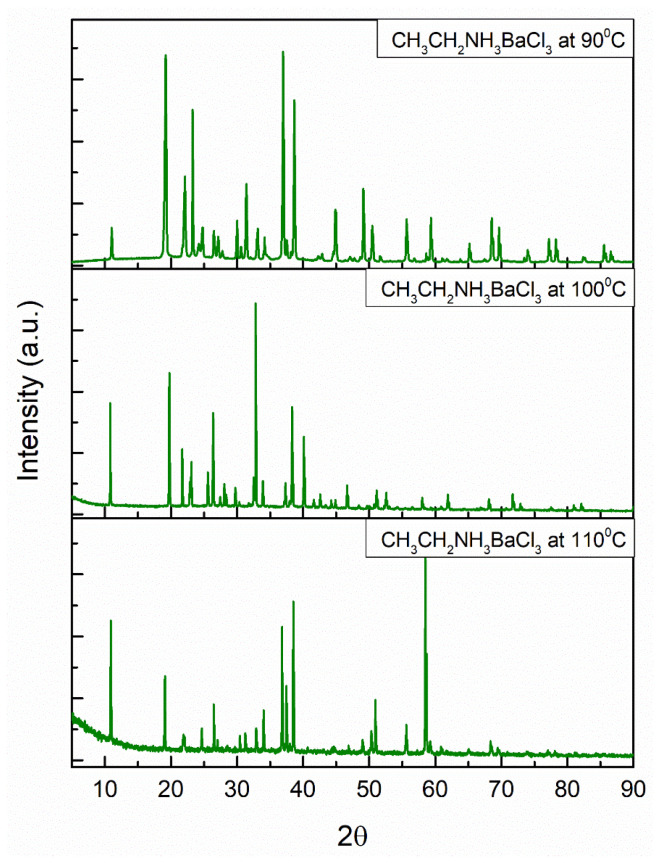
Experimental X-ray powder diffraction pattern of barium perovskite with an orthorhombic structure at 90, 100, and 110 °C.

**Figure 5:**
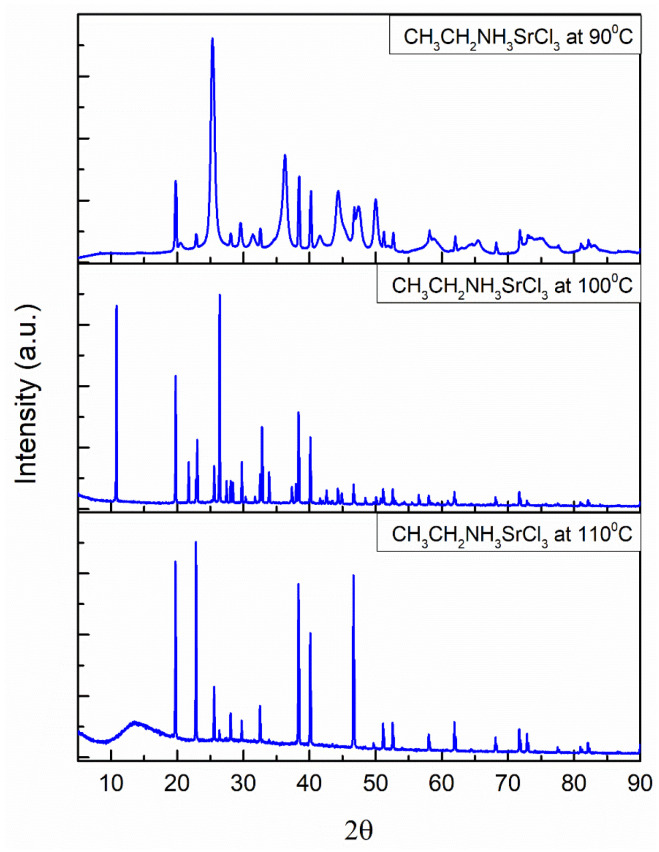
Experimental X-ray powder diffraction pattern of strontium perovskite with a tetragonal structure at 90, 100, and 110 °C.

Figure 4 and Figure 5 show the XRD patterns for the barium perovskite and strontium perovskite powders at 90, 100, and 110 °C. The narrow diffraction peaks indicate a long-range crystalline domain for the perovskite powders and a high crystallinity, except for strontium and barium perovskite at 90 °C, where broader peaks are shown compared to other perovskites at higher temperatures. This may be owed to the crystallization speed. Some peaks from XRD data have different intensities, although they are in the same position. The reason for this is the way perovskites deal with temperature, hence, the structure loses some of its symmetry with a gradual increase in temperature, which results in a variation in the height and intensity of the peaks in the XRD pattern.

Due to the synthesis method and the replacement of organic, metallic, and halide cations, the XRD pattern of these perovskites has not been reported in conventional databases. The cell parameters, volumes, space groups, and crystal systems of the perovskites are summarized in Table 1 and Table 2.

The EXPO2014 software was used to resolve the structures of the two perovskites, one consisting of strontium ethylamine strontium chloride (CH_3_CH_2_NH_3_SrCl_3_) and the other of ethylamine barium chloride (CH_3_CH_2_NH_3_BaCl_3_), using direct methods (DMs).

The diffraction patterns were processed to determine the new crystal structures and the positions of localized atoms in the structures, using comparisons with other perovskites containing lead, strontium methylamine chlorides, and iodides [19,49,50,51].

From the diffraction pattern, unique diffraction intensities were determined for each reflection in the 2θ range by EXPO2014, which established the shape and size of the unit cell. The angular positions of the diffraction lines and the spatial distribution of atoms within the cell were indicated by the relative intensity peaks or lines in the ethylamine barium and strontium chloride perovskites. The cell parameters, cell volume, space group, and reflection number were determined by the N-TREOR09, DICVOL06, and McMaille indexing processes to improve the indexing power and minimize errors [52]. The experimental resolution was 1.0894 Å for the six structures, and the space groups were P n c 2, P m c 21, and P 2 2 21 for the barium perovskites, with an orthorhombic structure, and P-4 21 m, P 42 21 2, and P-4 for the strontium perovskites, with a tetragonal structure.

The structures of the barium and strontium perovskites were first classified according to the CFOM, and only the best trial with the highest CFOM was used to calculate a Fourier map. There are three options for optimizing a structure model, as follows: (a) a resolution bias modification (RBM) algorithm [41]—this was used for the CH_3_CH_2_NH_3_BaCl_3_ perovskites at 100 °C and the CH_3_CH_2_NH_3_SrCl_3_ perovskite at 110 °C, which corresponded with choice representation for cases with organic and metal–organic compounds; (b) suitably weighted least squares (wLSQ) [40]—structures resolved by this method were the CH_3_CH_2_NH_3_BaCl_3_ perovskites at 90 °C and the CH_3_CH_2_NH_3_SrCl_3_ perovskites at 90 and 100 °C, and this procedure reduces errors caused by a limited experimental resolution in electron density maps; and (c) electron density modification, which is based on the covariance between map points (COVMAP) approach [42] and used for the CH_3_CH_2_NH_3_BaCl_3_ perovskite structure at 110 °C. At this point, the structural model provided by RBM was subjected to the COVMAP procedure, which appropriately modified the electron density map and submitted the enhanced model for wLSQ analysis.

### 3.3. Chemical Characterization

#### Chemical Characterization by FT-IR

**Figure 6:**
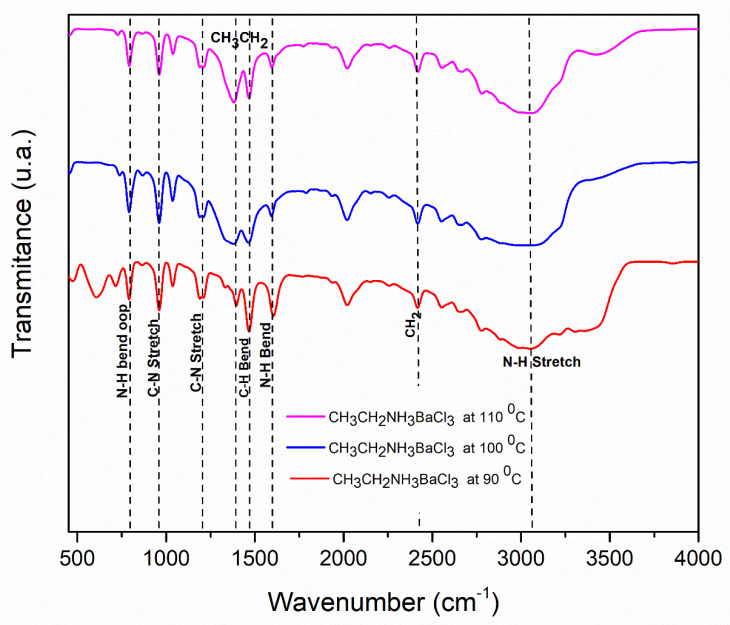
FTIR spectra for the barium perovskites (CH_3_CH_2_NH_3_BaCl_3_) at different temperatures.

The results of the Fourier transform infrared spectroscopy (FT-IR) analysis were employed to monitor the characteristics of the evolution of the chemical structure that the samples underwent upon thermal change. Barium perovskite is shown in Figure 6. The characteristic bonds containing the ethylamine (CH_3_CH_2_NH_3_) organic cation can be seen in the perovskite. The peaks at 1467 cm^−1^ and 1384 cm^−1^ were related to the C-H bending vibrations of the -CH_3_-and -CH_2_- groups. In addition, vibration bands near 2415 cm^−1^ were characteristic of molecules containing long alkyl chains, because these bands came from the symmetrical and asymmetric stretching vibrations of the -CH_2_- group.

Evidence of vibration bands in the N-H stretching ammonium groups is found at 3062 cm^−1^ and a bending vibration is present at approximately 1601 cm^−1^, along with C-N stretching bonds located at 962 cm^−1^ and 1198 cm^−1^. Out-of-plane bands belong to long-chain methyl rock at 789 cm^−1^, which is due to N-H bending wag for secondary amines.

**Figure 7:**
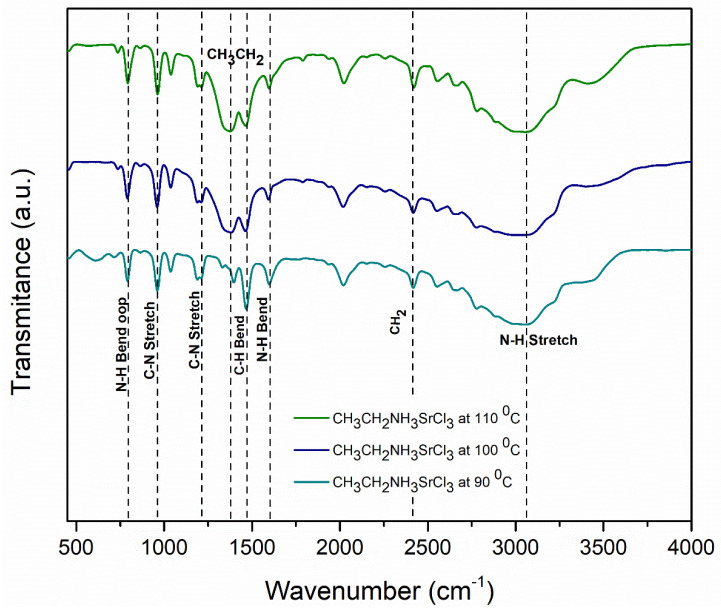
FTIR spectra for the strontium perovskites (CH_3_CH_2_NH_3_SrCl_3_) at different temperatures.

For the strontium ethylamine chloride perovskite, Figure 7 shows characteristic ethylamine N-H stretching bonds located around 3058 cm^−1^ and bending vibration at 1596 cm^−1^. The methyl and methylene functional groups have C-H bending bonds at 1466 cm^−1^ and 1395 cm^−1^. The stretching vibration bands of a methylene -CH_2_- group containing long alkyl chains is located at 2417 cm^−1^. The stretching of a C-N amine is located at 960 and 1211 cm^−1^, and bands out of the plane of the N-H bending wag are indicated at 790 cm^−1^.

It should be noted that there were not visible characteristics corresponding to stretching O-H vibration in the region of 3200–3500 cm^−1^, indicating the presence of a hydroxyl group in both perovskites and confirming the preservation of the crystal structure of the perovskites.

Upon thermal changes, there was a reduction in and widening of the C-H, N-H, and C-N bands from 90 to 110 °C in both perovskites, which may have been due to a change in the bonding of the Ba and Sr halide perovskites framework with ethylamine cation.

### 3.4. Photo-Electronic Properties

#### 3.4.1. Photoluminescence (PL)

**Figure 8:**
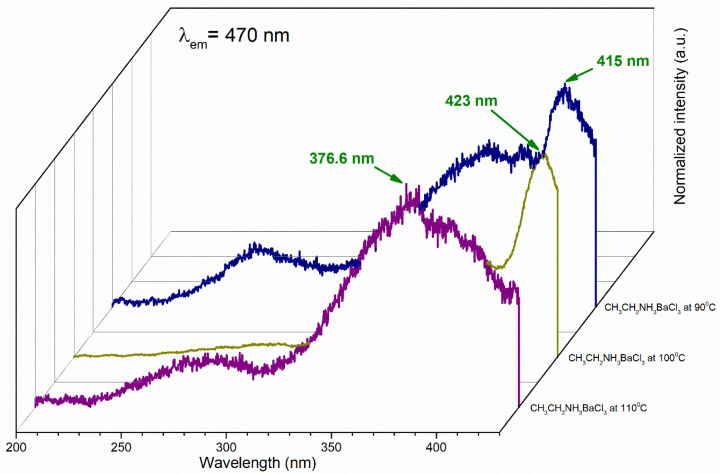
Absorption spectra of CH_3_CH_2_NH_3_BaCl_3_ at 90, 100, and 110 °C.

The barium ethylamine perovskites at 90, 100, and 110 °C absorbed light at 415 nm, 423.2 nm, and 376.6 nm, respectively (Figure 8). They exhibited a hypsochromic effect, as the absorption peaks tended to shift to shorter wavelengths and absorb violet color (390–430 nm), except for the perovskite at 110 °C, which absorbed light in the near-UV range and exhibited an n-π* transition (200–700 nm) [53].

**Figure 9:**
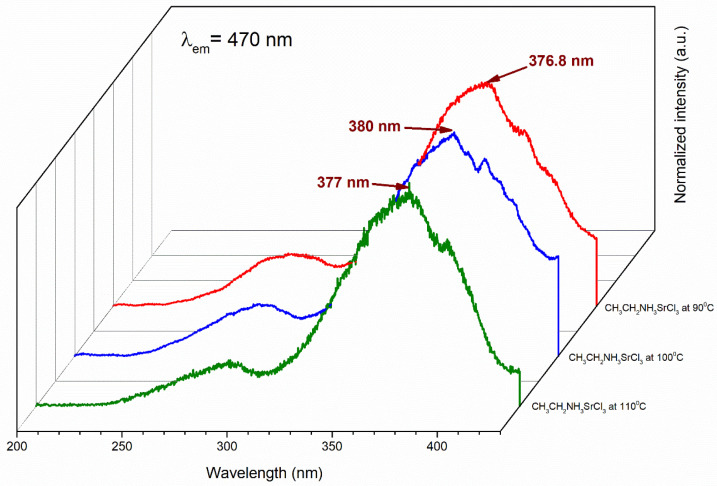
Absorption spectra of CH_3_CH_2_NH_3_SrCl_3_ at 90, 100, and 110 °C.

This occurred because, in molecules with n-π* transitions, unshared electrons form H bridges with H^+^ from the organic solvent; hence, the transition requires more energy. The band gap of the cation halogen salt (ethylamine chloride (CH_3_CH_2_NH_3_Cl^−^)) showed that the highest occupied molecular orbital (HOMO) was delocalized in the chlorine ion and descended to the lowest molecular orbital (LUMO), distributing around the barium and strontium metallic cation, which corresponded with a lower energy jump. Typically, the electron was excited and was of a considerable size where absorption occurred in the violet and near-UV regions in the electromagnetic spectrum; therefore, the η-π* transition occurred at a shorter wavelength, evident in the barium perovskites. Generally, η-π* transitions were weaker (i.e., absorbed less light) than π-π* transitions [54,55,56]. For the strontium perovskites in Figure 9, absorption also occurred in the near-UV region, showing a hypsochromic shift.

**Figure 10:**
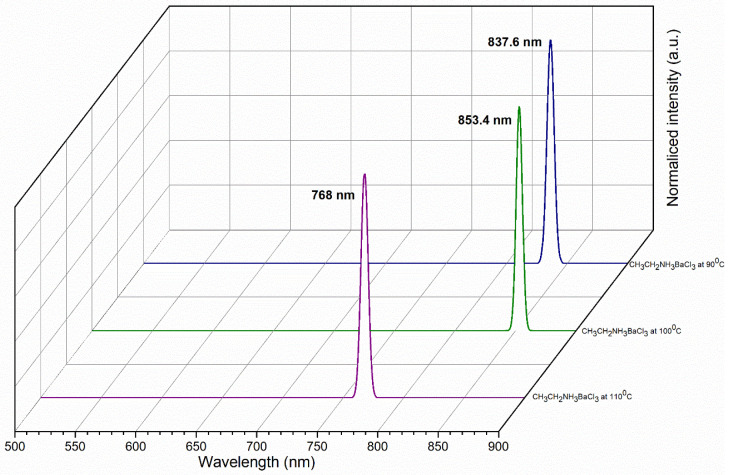
PL emission spectra of CH_3_CH_2_NH_3_BaCl_3_ at 90, 100, and 110 °C.

The barium perovskite had a photoluminescence (PL) infrared emission peak at 837.6 nm and 853.4 nm at 90 °C and 100 °C. At 110 °C, the barium perovskite had a PL peak at 768 nm (Figure 10), and exhibited a redshift as the temperature increased, which corresponded to a band-to-band recombination of electrons in the conduction band with holes in the valence band. The most apparent change that can be seen from the PL spectra is that, in perovskite at 100 °C, an increase in the luminescent center concentration should be accompanied by an increase in the intensity of emitted light due to a higher absorption efficiency [57].

This is due to the charge carriers gaining mobility as the temperature increases and, therefore, becoming increasingly able to reach the non-radiative recombination defects present in perovskites. This leads to an increase in the quantum yield of photoluminescence at this temperature and can, instead, recombine radiatively.

**Figure 11:**
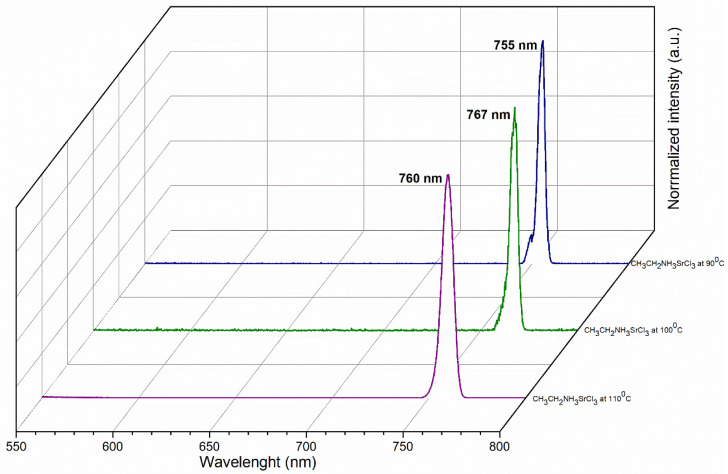
PL emission spectra of CH_3_CH_2_NH_3_SrCl_3_ at 90, 100, and 110 °C.

In the strontium perovskite, the emission peaks are observed in a red color with a PL wavelength to 755 nm, 767 nm, and 760 nm, which correspond to the temperatures of 90, 100, and 110 °C (Figure 11) and are close to the infrared zone. The PL also shifts with temperature, increasing at the beginning and then decreasing at higher temperatures. The PL intensities of strontium perovskite are lower than those of barium perovskite, indicating a more radiative recombination in the barium perovskite [58].

In barium and strontium perovskites, it can be seen that the PL wavelength does decrease with increasing temperature. This is because the crystal structures have different band gaps due to the different orbital overlaps within the phases, and the PL wavelength changes.

#### 3.4.2. UV–Vis Spectroscopy

**Figure 12:**
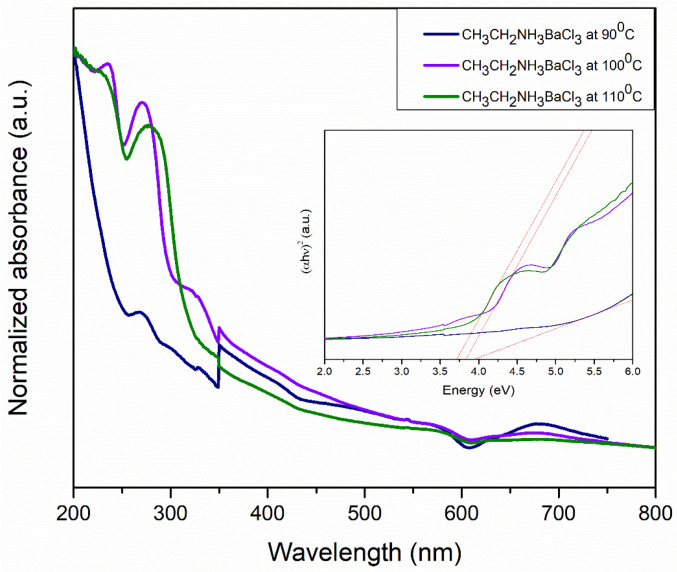
Normalized UV–Visible spectra of CH_3_CH_2_NH_3_BaCl_3_ perovskite at 90, 100, and 110 °C. Inset: corresponding Tauc plots.

The absorption spectra of barium perovskite at 90, 100, and 110 °C are shown in Figure 12. Plotting the (αhv)γ versus (hv) is a matter of testing γ =2 r for indirect allowed transitions. If we compare Tauc’s equation with the straight-line equation, putting the y-axis equal to zero will bring us the x-axis. And the solving for the energy, the extrapolation of the linear region of the plot onto the x-axis, gives the band gap or edge energy. The band gap determined through Tauc plot (αhν)2 analysis resulted in E_g_ values of 3.93, 3.63, and 3.67 eV at the three different temperatures. 

These perovskites showed a shift towards the near-UV region, although, as the temperature increased, there was a decrease in the band gap.

**Figure 13:**
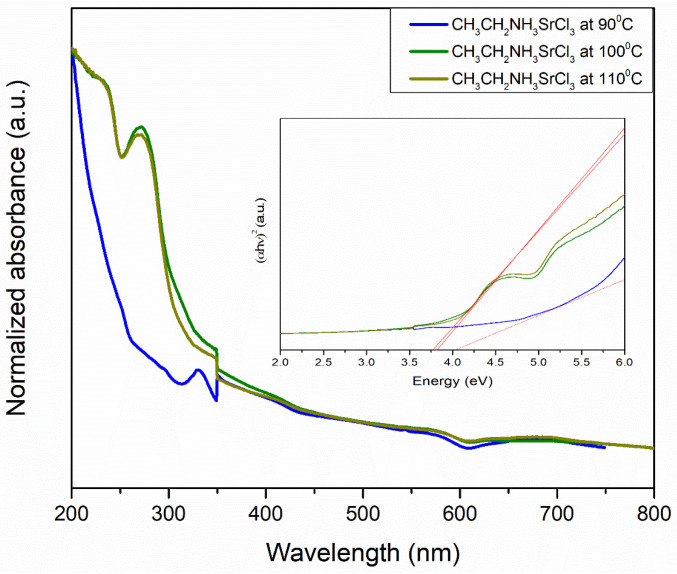
Normalized UV–Visible spectra of CH_3_CH_2_NH_3_SrCl_3_ perovskite at 90, 100, and 110 °C. Inset: corresponding Tauc plots.

For strontium perovskite at 90, 100, and 110 °C, the E_g_ values were 4.05, 3.76, and 3.84 eV, respectively (Figure 13). These E_g_ intervals, along with those of the barium perovskite, did not fit with the band gap values for a photo-absorbing compound (<1.5 eV), so from the perspective of its use as a replacement material for lead in lead-based perovskite cells, its prospects are remote; however, it may have other applications, such as in photocatalysis, UV filters, electron transport materials in tandem cells, or as a transparent contact layer [59,60].

The top of the valence band is composed mainly of energy levels that originate at the upper edge of the valence band dominated by chlorine orbitals (3p^5^), or more specifically, by 6s^2^ (for barium perovskite) and 5s^2^ (for strontium perovskite) electrons. Since they contribute to the lower edge of the conduction band, these energy levels are lower than the Pb 6p orbitals; therefore, the valence band does not change. The makeup of the lower part of the conduction band is similar for the two perovskites. This is because the energy states at the lower part of the conduction band stem from extensive hybridization among 6s, 5s, and Cl 5p orbitals; ethylamine also contributes to the states at the bottom of the conduction band [61]. The change in the edge of the conduction band in the strontium and barium perovskites is large enough for the electronic properties of the organic ion to have a more considerable influence on the band gap, but this does not occur due to its low electronegativity. Hence, the electronegativity of both alkaline earth elements (Sr = 0.95 and Ba = 0.85), compared to that of lead cells, displaces the electron cloud closer to the chlorine atoms in the lattice, which partly explains this effect. This also influences the E_g_ values, as it decreases with increasing electronegativity of cation B, such as lead (2.33); therefore, the electronegativity of cation B has a greater impact on the band gap than cation A (the ethylamine cation).

**Figure 14:**
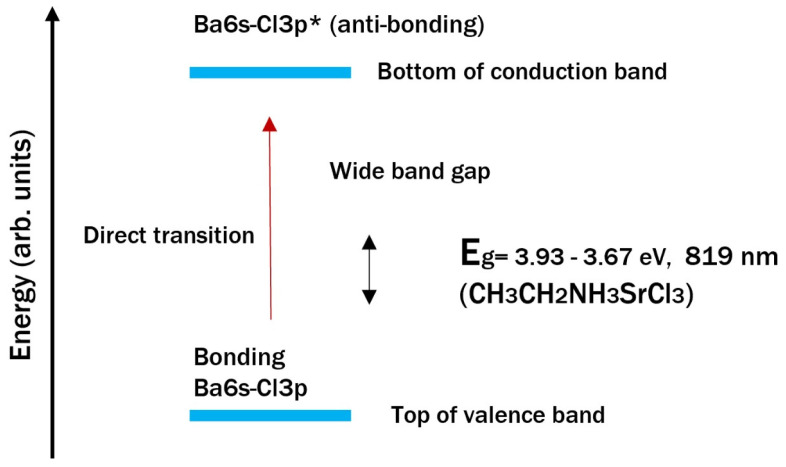
Band gap structure and energy levels of barium perovskite.

**Figure 15:**
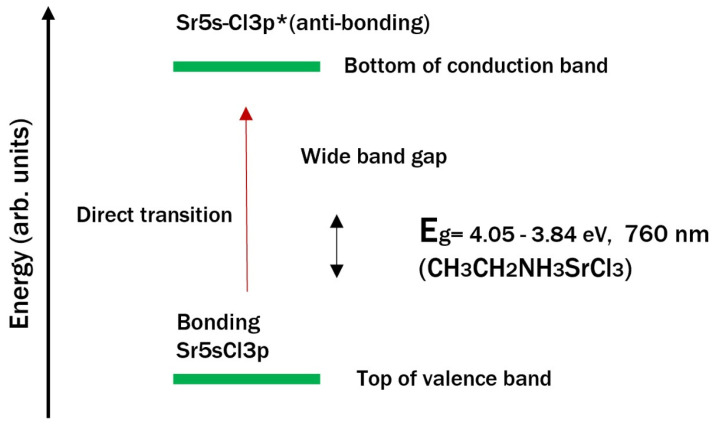
Band gap structure and energy levels of strontium perovskite.

Figure 14 and Figure 15 show the band structures of the barium and strontium perovskites. The valence band (VB) of CH_3_CH_2_NH_3_BaCl_3_ consists of mostly Cl 3p orbitals and the rest of the Ba 6s2 (lone pair) orbitals, whereas the conduction band (CB) consists of a mixture of Ba 6s and other orbitals. The valence band orbitals have a strong coupling between Ba 6s2 and Cl 3p lone pair orbitals. In Figure 14, Cl 3p orbitals are also predominant in the valence band, and the conduction band possesses Sr 5s^2^ orbitals. The high symmetry and direct band gap of CH_3_CH_2_NH_3_BaCl_3_ and the p−s electron transitions from the valence band to the conduction band, enabled by the lone orbital pair of Ba, contribute to slightly higher optical absorption coefficients than those of strontium.

The defective perovskite properties are attributed to a strong antibonding coupling of Ba and Sr s−Cl p. A weak antibonding coupling between Ba, Sr s, and Cl p orbitals sets the conduction band minimum (CBM) near the s orbital of metal cations.

The high ionic density in chloride halide perovskites is also considered to help suppress electron–hole recombination by a charge filter effect against the Coulombic interaction [62,63,64].

## 4. Conclusions

This study explored a new method for discovering and evaluating alternative elements that could replace the lead in traditional lead-based perovskites for potential use in solar cells. Substitutions for the metallic cation (Ba and Sr), the organic cation (ethylamine), and the anion (Cl^−^) affected the band gap of the perovskites. The results from the excitation spectra showed high optical absorption only in the near-ultraviolet range (200–400 nm) and only partially in the visible region (absorbing violet light at 380–420 nm), with a trend towards shorter wavelengths (blue shift), resulting in n-π* electronic transitions for both perovskites. The PL wavelength was found to redshift with an increasing temperature, indicating that the bandgap of Ba and Sr perovskites decreased as the temperature increased.

This achievement combines crystal chemistry with the availability of elements that have the potential to take the place of lead because of their similar ionic radii and charges. The resolution of the structures of the perovskite samples using the EXPO2014 software revealed tetragonal cells for the strontium perovskites and orthorhombic cells for the barium perovskites. The structural resolution utilized direct methods and a structural optimization model using the RBM, wLQS, and COVMAP algorithms for both perovskites.

This study focused on barium (Ba^2+^ 149 pm) and strontium (Sr^2+^ 132 pm) because their ionic radii are close to that of lead (Pb^2+^ 133 pm), so they could be substituted for lead while maintaining a stable perovskite structure. Structural resolution calculations by the EXPO2014 software indicated that the strontium and barium perovskites exhibited stable phases and cell structures like some lead-based perovskites, for example, the tetragonal structures observed in MAPbCl-x perovskites.

The band gap of the strontium perovskites ranged from 4.05 to 3.84 eV; the band gap of the barium perovskites ranged from 3.93 to 3.67 eV. These values were associated with increases in temperature, which controlled phase formation and could lead to distortions in the crystal lattice and potential changes in the unit cell, which were reflected in the band gap; in this case, these were significantly higher than those in lead perovskites (CH_3_NH_3_PbCl_3_ 1.16 eV).

It is concluded that the ideal temperature to synthesize barium and strontium perovskite is 100 °C, since this obtained the major absorption and emission wavelength, had the lowest value in band gap, and was the best temperature for the stable formation of orthorhombic and tetragonal phases.

In sum, the band gaps of strontium and barium elements were comparatively higher than those of lead, suggesting that they may not be suitable for direct application in solar cells; nevertheless, lowering them by substituting other metals with a higher electronegativity could lead to interesting possibilities, such as tandem and multijunction solar cell light-emitting diodes (LEDs), indoor lighting, and photodetectors.

## Figures and Tables

**Table 1:** Crystallographic information. The structural parameters of the tetragonal crystalline system for the strontium perovskites. All the columns report the results from direct methods resolution by EXPO2014.

Cell Content	Cell Parameters	$\mathrm{Cell}\;\mathrm{Volume}\;({\dot{A}}^{3})$	Space Group	Number of Reflections NR	$\mathrm{Experimental}\;\mathrm{Resolution}\;d(\dot{A})$	Crystalline System
CH_3_CH_2_NH_3_SrCl_3_ at 90 °C	$a=7.010\;(\dot{A})$ $b=7.010\;(\dot{A})$ $c=10.504\;(\dot{A})$ α = 90.00 β = 90.00 γ = 90.00	515.4	P-4 21 m	159	1.089	Tetragonal
CH_3_CH_2_NH_3_SrCl_3_ at 100 °C	$a=13.095\;(\dot{A})$ $b=13.095\;(\dot{A})$ $c=20.938\;(\dot{A})$ α = 90.00 β = 90.00 γ = 90.00	358.5	P 42 21 2	900	1.089	Tetragonal
CH_3_CH_2_NH_3_SrCl_3_ at 110 °C	$a=5.502\;(\dot{A})$ $b=5.502\;(\dot{A})$ $c=4.492\;(\dot{A})$ α = 90.00 β = 90.00 γ = 90.00	135.48	P-4	62	1.089	Tetragonal

**Table 2:** Crystallographic information. The structural parameters of the orthorhombic crystalline system for the barium perovskites. All the columns report the results from the direct methods resolution in EXPO2014.

Cell Content	Cell Parameters	$\mathrm{Cell}\;\mathrm{Volume}\;({\dot{A}}^{3})$	Space Group	Number of Reflections NR	$\mathrm{Experimental}\;\mathrm{Resolution}\;d(\dot{A})$	Crystalline System
CH_3_CH_2_NH_3_BaCl_3_ at 90 °C	a $=12.029\;(\dot{A})$ b = $16.363\;(\dot{A})$ c = $2.927\;(\dot{A})$ α = 90.00 β = 90.00 γ = 90.00	577.6	P n c 2	396	1.08944	Orthorhombic
CH_3_CH_2_NH_3_BaCl_3_ at 100 °C	a = $4.496\;(\dot{A})$ b = $8.188\;(\dot{A})$ c = $7.439\;(\dot{A})$ α = 90.00 β = 90.00 γ = 90.00	273.6	P m c 21	156	1.08944	Orthorhombic
CH_3_CH_2_NH_3_BaCl_3_ at 110 °C	a $=13.931\;(\dot{A})$ b $=5.562\;(\dot{A})$ c $=9.901\;(\dot{A})$ α = 90.00 β = 90.00 γ = 90.00	766.87	P 2 2 21	407	1.08944	Orthorhombic

## Data Availability

The data that support the findings of this study are available on request from the corresponding author. The data are not publicly available due to privacy or ethical restrictions.
